# Decomposition of overlapping protein complexes: A graph theoretical method for analyzing static and dynamic protein associations

**DOI:** 10.1186/1748-7188-1-7

**Published:** 2006-04-26

**Authors:** Elena Zotenko, Katia S Guimarães, Raja Jothi, Teresa M Przytycka

**Affiliations:** 1 National Center for Biotechnology Information, National Library of Medicine, National Institutes of Health, Bethesda, USA; 2 Department of Computer Science, University of Maryland, College Park, USA; 3 Center of Informatics, Federal University of Pernambuco, Recife, Brazil

## Abstract

**Background:**

Most cellular processes are carried out by multi-protein complexes, groups of proteins that bind together to perform a specific task. Some proteins form stable complexes, while other proteins form transient associations and are part of several complexes at different stages of a cellular process. A better understanding of this higher-order organization of proteins into overlapping complexes is an important step towards unveiling functional and evolutionary mechanisms behind biological networks.

**Results:**

We propose a new method for identifying and representing overlapping protein complexes (or larger units called *functional groups*) within a protein interaction network. We develop a graph-theoretical framework that enables automatic construction of such representation. We illustrate the effectiveness of our method by applying it to TNF*α*/NF-*κ*B and pheromone signaling pathways.

**Conclusion:**

The proposed representation helps in understanding the transitions between functional groups and allows for tracking a protein's path through a cascade of functional groups. Therefore, depending on the nature of the network, our representation is capable of elucidating temporal relations between functional groups. Our results show that the proposed method opens a new avenue for the analysis of protein interaction networks.

## Background

A major challenge in systems biology is to understand the intricate network of interacting molecules. The complexity in biological systems arises not only from various individual protein molecules but also from their organization into systems with numerous interacting partners. In fact, most cellular processes are carried out by multi-protein complexes, groups of proteins that bind together to perform a specific task. Some proteins form stable complexes, such as the ribosomal complex that consists of more than 50 proteins and three RNA molecules, while other proteins form transient associations and are part of several complexes at different stages of a cellular process. A better understanding of this higher-order organization of proteins into overlapping complexes is an important step towards unveiling functional and evolutionary mechanisms behind biological networks.

Data on protein complexes are collected from the study of individual systems, and more recently through high-throughput experiments, such as yeast two-hybrid (Y2H) [1,2] and tandem affinity purification followed by mass spectrometry (TAP/MS) [3,4]. The TAP/MS approach helps pinpoint proteins that interact with a tagged *bait *protein, either directly or indirectly, and are thus suited to identify multi-protein complexes. In fact, several research groups have systematically applied TAP/MS technology to study protein complexes involved in different signaling pathways [5].

Protein interactions are routinely represented as graphs, with proteins as nodes and interactions as edges (links). Therefore, it is not surprising that analysis of protein interaction networks reach out for a variety of graph-theoretical tools. Following the observation that protein interaction networks display a characteristic power-law like node degree distribution [6], a substantial body of research focused on statistical properties of protein interaction networks [7,8]. In 1999, Hartwell *et al. *[9] introduced a notion of a *functional module*, a group of cellular components and their interaction that can be attributed a specific biological function. The authors also suggested the modular organization of molecular interaction networks, where each functional module involves a small number of cellular components and is autonomous, i.e., its interaction with other modules is limited to a few cellular components. Subsequently, this assumption was used in several computational methods to identify protein complexes and functional modules in high-throughput protein interaction networks [10-15]. Some methods [10-13] look for densely connected subgraphs within a protein interaction network, either *cliques *or *"cliquish" *components. For example, Spirin *et al. *[13] use the term functional module to denote groups of proteins which are densely connected within themselves but sparsely connected with the rest of the network. Other methods [14,15] combine protein interaction with other information to identify functional modules, such as signal transduction pathways, that do not necessarily correspond to densely connected regions of the network. 

In a recent paper, Gagneur *et al. *applied *modular decomposition *to elucidate the organization of protein complexes [16]. The basic principle behind modular decomposition is to iteratively identify and contract nodes that are in a certain sense equivalent, until no more equivalent nodes can be found in the graph. A graph is called *prime *if it cannot be decomposed any further. Only graphs that belong to a very special graph family called *cographs *can be completely decomposed (that is, the iterative reduction process does not halt with a non-trivial prime graph). While the modular decomposition provides an excellent description of combinatorial variants within a family of complexes, it does not impose any order on the complexes within the family. As such it lacks the description power to represent the dynamics of complex formation, i.e., the manner in which proteins form transient interactions to participate in the complexes within the family. The order imposed on protein complexes within the family is particularly interesting if the family corresponds to a functional module where biological function is achieved through a dynamic formation of protein complexes and the order reflects this formation.

In this work, we model a functional module as a union of overlapping dense subnetworks called here *functional groups*. A functional group is either a maximal clique (typically representing a protein complex) or a set of alternative variants of such complexes/cliques. As components of a larger functional module, functional groups are not assumed to be well separated and can have significant overlaps. Intuitively, if a functional module performs a function that requires a sequence of steps (like in the case of a signaling pathway) then we would like functional groups to be snapshots of protein associations at these steps. We propose a new method for identifying and representing overlapping functional groups in a functional module. Furthermore, if the module corresponds to a dynamic process that requires certain complexes (or more generally functional groups) come into contact in a specific order, our method attempts to discover this order. Our method is motivated by a fundamental result for *chordal graphs *[17], which states that every chordal graph has the so called *clique tree representation*. However, not every protein interaction network is chordal and not every functional group is a clique. Therefore, we developed a graph-theoretical framework that enables automatic construction of a tree-like representation, analogous to the clique tree representation, for much broader family of graphs. We call this representation the *Tree of Complexes *representation. The nodes in the tree are functional groups, and for every protein, the set of functional groups that contain this protein forms a single subtree. The "single subtree" requirement restricts significantly the way in which the nodes of the tree can be interconnected. As a consequence, this representation shows a smooth transition between functional groups and allows for tracking a protein's path through a cascade of functional groups. Therefore, depending on the nature of the network, the representation may be capable of elucidating temporal relations between functional groups.

We developed a new method, *Complex Overlap Decomposition *(COD), that given a protein interaction network identifies its functional groups and constructs the Tree of Complexes representation. Our method requires that the network satisfies certain mathematical properties. We applied the COD method to several protein interaction networks, such as the TNF*α*/NF-*κ*B signaling pathway and the pheromone signaling pathway. The corresponding subnetworks for all interaction networks are extracted from high throughput experimental data. Our results show that the COD method opens a new avenue for the analysis of protein interaction networks.

## Results and discussion

One way to represent a set of overlapping functional groups is to construct a graph with nodes representing functional groups and edges representing overlaps, i.e., there exists an edge between two functional groups if and only if they share at least one protein. This approach has two shortcomings. First, it is not obvious how to correctly identify functional groups, and second, such a representation does not provide any information about the dynamics of proteins in the network. We propose a graph-theoretical approach, which, under the assumption that the protein interaction network satisfies certain mathematical properties, identifies functional groups and provides a representation of overlaps between functional groups in the form of the Tree of Complexes.

Here, we first describe the COD method. Then, we demonstrate the utility of our approach by applying the COD method to several examples, derived from high-throughput experiments, TNF*α*/NF-*κ*B and pheromone signaling pathway interaction networks.

### Complex overlap decomposition

**Figure 1:**
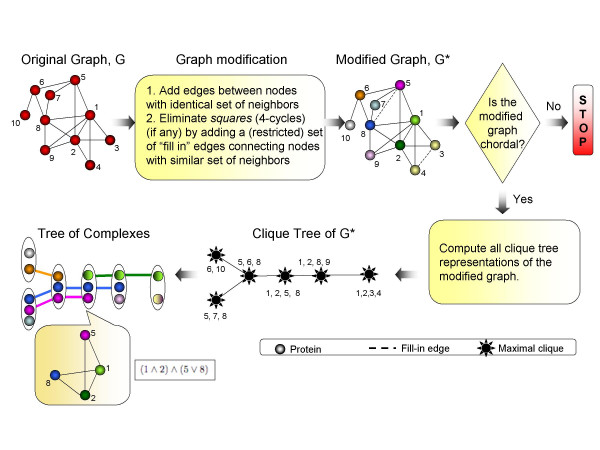
**Complex Overlap Decomposition**. A simplified illustration of the Complex Overlap Decomposition (COD) method. An edge, (3, 4), connecting a pair of weak siblings is added to the graph. A fill-in edge between proteins 5 and 8 is added to eliminate all five 4-cycles in the graph: {5, 6, 8, 7}, {1, 5, 7, 8}, {2, 5, 7, 8}, {1, 5, 6, 8}, and {2, 5, 6, 8}. If the modified graph is chordal, all clique tree representations are computed (cf. Methods). Each clique tree representation results in a Tree of Complexes representation, where the Tree of Complexes is constructed by projecting each maximal clique in the modified graph, G*, to a functional group in the original graph *G*. For example, a four node maximal clique, {1, 2, 5, 8}, in G* is projected to a four node functional group in *G*, by removing a fill-in edge (5, 8). Each functional group is represented by a Boolean expression, such as (1 ∧ 2) ∧ (5 ∨ 8), which means that the functional group contains two variants of a complex, {1, 2, 5} and {1, 2, 8}.

Our method of representing overlapping functional groups, which is depicted in Figure 1, builds on chordal and cograph graph theories. Chordal graphs constitute an important and well studied graph family [18,19]. A *chord *in a graph is any edge that connects two non-consecutive nodes of a cycle. A *chordal graph *is a graph which does not contain chordless cycles of length greater than three.

An important property of chordal graphs, which is explored directly in this paper, is that every chordal graph has a corresponding *clique tree representation or clique tree *[17]. The nodes in the tree are maximal cliques. Moreover, for every node in the graph, a set of maximal cliques that contain this node form a connected subgraph of the clique tree. Thus, there is a mapping between the nodes in the graph and subtrees in the clique tree. The "connected subgraph" requirement puts constraints on the topology of the clique tree. In fact, the topology of the tree is determined by the structure of overlaps between the maximal cliques in the graph. Thus, the clique tree captures information about the structure of the overlaps, which is lost in a simple clique intersection graph as shown in the example below.

**Figure 2:**
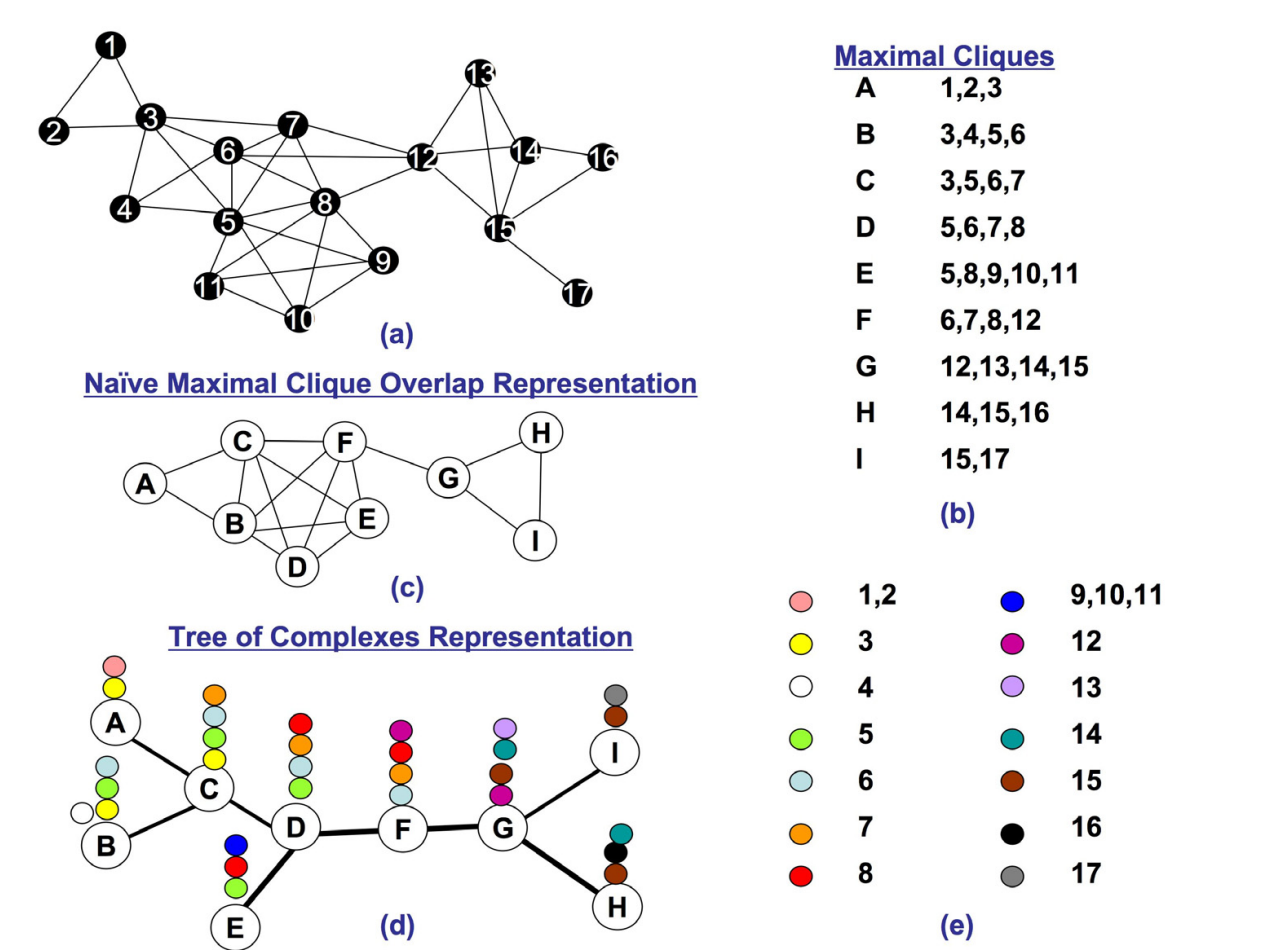
**A Hypothetical Protein Interaction Network**. (a) A hypothetical protein interaction network. (b) A list of all maximal cliques in the network. (c) A naive representation of overlaps between maximal cliques. Each maximal clique is a node and there is an edge between two maximal cliques if and only if they share a protein. (d) The clique tree representation. Once again, every maximal clique is a node, but the cliques are connected in such a way that the resulting graph is a tree. Moreover, cliques that contain a given protein form a connected subgraph. (e) This color scheme is used to show the subtree of every protein. For example, protein 3 is contained in maximal cliques *A, B*, and *C*, which is shown by placing yellow dots above the maximal cliques.

**Example **Consider a hypothetical protein interaction network in Figure 2(a). This network is chordal and its maximal cliques are listed in Figure 2(b). We want to contrast the clique tree representation in Figure 2(d) to a naive representation in Figure 2(c), where every pair of maximal cliques that contain a protein in common is connected by an edge.

While both representations show the overlap between maximal cliques, the interconnection pattern of cliques in the naive representation carries little additional information about the structure of this overlap. On the other hand, a very specific tree-like interconnection pattern in the clique tree representation can expose a special structure of such overlap. For example, consider maximal cliques *B *through *F*. In the naive representation, the overlap between these maximal cliques is collapsed to a clique. Thus, the representation treats the maximal cliques and overlaps between them equally. In particular, there is no way to tell that, for example, *D *occupies a more central position in the network than *B*. In the clique tree representation this information can be extracted from the relative position of cliques in the tree. For example, *B *is connected to *F *by a path that passes through *C *and *D*, which means that any protein shared by *B *and *F *is also contained in *C *and *D*. In other words, the overlap between *B *and *F *is entirely contained in the overlap between *B *and *D*, which in turn is entirely contained in the overlap between *B *and *C*. Thus, there is a correlation between the amount of overlap between maximal cliques and their distance in the clique tree.

Nice properties of the clique tree mentioned above make it a good choice for representation of overlaps between functional groups. However, not every protein interaction network is chordal and maximal cliques may not always be the best way to represent functional groups. For example, in Figure 1, cliques {1, 2, 3} and {1, 2, 4} may correspond to two variants of one complex, where proteins 3 and 4 replace each other, forming one rather than two functional groups. Therefore, in the COD decomposition we relax the assumption that every functional group is a single protein complex (maximal clique) and allow it to contain several protein complexes (maximal cliques). In doing so we have to ensure that a functional group is not just any collection of protein complexes but rather a set of closely related protein complexes which represent possible variants of one complex (such as complexes {1, 2, 3} and {1, 2, 4} in the above example). To capture this systematically we model a functional group with a graph from a family of graphs known as cographs.

Cographs are another well-studied graph family [20]. A *cograph *can be characterized by an absence of an induced subgraph which is a path of length four (*P*_4_), where the length is the number of nodes in the path. Thus, the diameter of a connected cograph is at most two. Subsequently, connected cographs are dense and cliquish, consistently with the assumption made by algorithms that delineate protein complexes. What makes cographs even more attractive is that for every cograph there exists a Boolean expression which describes all the maximal cliques in the graph. (In terms of modular decomposition used in [16] it means that a cograph can be decomposed by modular decomposition without leaving non-trivial non-decomposable prime module.) This Booolean expression describes in a compact and hierarchical way all the possible variants of protein complex within a functional group.

The main idea behind COD method is to provide a representation of a functional module, which is analogous to the clique tree, in which nodes are cographs (representing variants of protein complex within a functional group) rather than maximal cliques. If we knew in advance all the functional groups in the module, we could simply connect the proteins within each functional group turning it into a clique and, under the assumption that the resulting graph is chordal, apply clique tree construction algorithm to the graph. Since we do not have predefined functional groups, our algorithm identifies them by adding edges to the graph in such a way that each added edge connects a pair of nodes that putatively belong to the same functional group.

The COD method's edge addition strategy and its biological motivation builds on a concept of *weak siblings*. We call a pair of nodes weak siblings if and only if they are connected to the exactly the same set of neighbors, but are not connected to each other. In terms of protein interaction networks, weak siblings are proteins which interact with the same set of proteins but do not interact with each other. In particular, proteins that can substitute each other in a protein interaction network may have this property. Similarly, a pair of proteins that belong to the same complex but are not connected due to missing data or an experimental error will be represented as weak siblings. Since the weak siblings relationship suggests functional similarity, the COD method takes a first step towards delineation of functional groups by connecting every pair of weak siblings by an edge. As this modification may also eliminate some of the chordless cycles of length four (*squares*) in the graph, functional group delineation happens simultaneously with transformation of the protein interaction graph into a chordal graph.

If, after connecting all pairs of weak siblings, the resulting graph is not chordal, the COD method attempts to transform it to chordal by adding some additional edges. Consistently with our assumption that we connect only nodes corresponding to proteins that could be put in the same functional group, we impose restrictions on this "fill-in" process. Namely, we require that, each introduced edge connects a pair of nodes which are close to being weak siblings. In such case the new edge is a diagonal of one or more squares in the graph. We emphasize that adding edges between nodes of longer cycles has no such justification. To summarize our edge addition procedure, our method attempts to eliminate all the squares in the protein interaction network by adding a limited set of diagonals that satisfies following conditions (i) connects potentially functionally equivalent proteins, as measured by the overlap in neighborhoods or distance from being a pair of weak siblings; (ii) ensures that functional groups correspond to cographs; we argue that this condition is guaranteed if the set of added edges does not form a *P*_4 _in a maximal clique of the modified graph (cf. Methods);

If the modification step succeeds, i.e., the modified graph is chordal, all the clique tree representations of the modified graph are computed and then each clique tree is extended to a Tree of Complexes representation of the original graph. The COD algorithm keeps track of all the edge additions and uses this information to delineate functional groups by projecting each maximal clique onto original network and removing all introduced edges contained in the clique. For example, in the modified graph of Figure 1 a maximal clique with four nodes, {1, 2, 5, 8}, is projected to a functional group by removing an edge connecting proteins 5 and 8. This functional group contains two variants of a protein complex, {1, 2, 5} and {1, 2, 8}, which are compactly represented by a (1 ∧ 2) ∧ (5 ∨ 8) Boolean expression. If, on the other hand, the modified graph is not chordal, the COD method stops without producing the representation. Since the clique tree representation for a chordal graph is not unique, the Tree of Complexes representation that derives from it is not unique either. As all clique trees of a chordal graph have the same set of nodes (the nodes are the maximal cliques in the graph), the difference between clique trees comes from the topology of the tree. The clique tree topology is determined by the "connected subgraph" constraints and restriction power of these constraints depends on the structure of the underlying graph, i.e., there are graphs with a unique clique tree representation and there are graphs for which almost any tree that spans all the maximal cliques in the graph is a valid clique tree. As a result a protein interaction network may have several Tree of Complexes representations; all such representations will have the same functional groups but will differ in the way these functional groups are interconnected. For every protein interaction network analyzed bellow we explicitly state all the possible Tree of Complexes representations.

### TNF*α*/NF-*κ*B signaling pathway

To illustrate the power of COD in elucidating the dynamics behind protein complexes, we consider the TNF*α*/NF-*κ*B signaling pathway. The Nuclear Factor *κ*B (NF-*κ*B) family of transcription factors is activated in response to a diverse set of stress stimuli, which includes pro-inflammatory cytokines, *e.g.*, TNF*α*. In vertebrates, this family includes p50, p52, Rel A, c-Rel, and Rel B, which bind to the DNA in a homo or heterodimeric fashion. The NF-*κ*B activity is regulated by the I*κ*B family of proteins via inhibitory ankyrin repeat domains. This family includes I*κ*B*α*, I*κ*B*β*, and I*κ*B*∈*. The precursors of p50 (p105) and p52 (pl00) also possess ankyrin repeat domains and thus act as inhibitors. These precursors can also form dimers with other members of the NF-*κ*B family. The activation with the pro-inflammatory cytokine tumor necrosis factor TNF*α *triggers a signaling cascade, which, in particular, stimulates the activation of the IKK*α*, IKK*β*, and IKK*γ *functional groups. The IKKs initiate a signal induced degradation of the inhibitors (I*κ*Bs), and subsequent nuclear translocation of the transcription factor. Recent TAP experiments [5] provide a wealth of new information regarding this important signaling pathway. Bouwmeester *et al. *identified 221 molecular associations, out of which only 80 where previously known. Gagneur *et al. *[16] applied modular decomposition to the network of these associations but the decomposition halted quickly at large non-decomposable modules.

**Figure 3:**
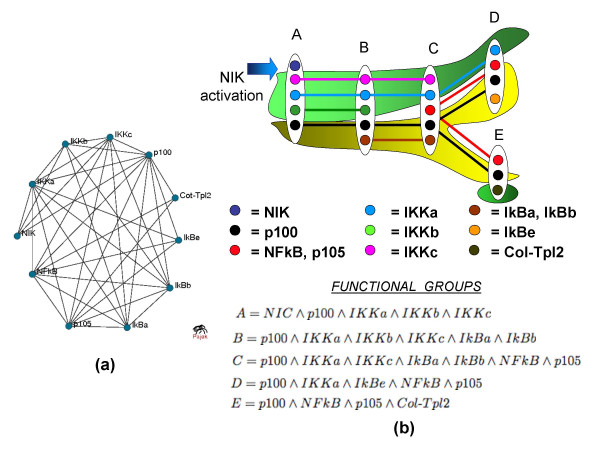
**TNF*α*/NF-*κ*B Signaling Pathway**. The TNF*α*/NF-*κ*B signaling pathway. (a) The network. (b) The Tree of Complexes representation. The flow of action is visually represented by background colors: green for activators (IKKs) and yellow for inhibitors (I*κ*Bs, and p100). The NIK kinase is in the first functional group (A), together with all three members of the IKK complex and p100. Functional group B includes, in addition to p100, the IKKs and two inhibitors I*κ*B*α *and I*κ*B*β*. This group is the beginning of interaction between IKKs and I*κ*Bs. Functional group C loses some of the IKKs, continues to show I*κ*B and begins to show interaction between I*κ*Bs and NF-*κ*B factors. Finally, in group E we see the entrance of NIK-independent Col-Tpl2 kinase.

We used the COD method to analyze the subnetwork spanning all the paths from NIK (NF*κ*B-inducing kinase phosphorylating IKK*α *and IKK*β*) with at most three edges. For the purpose of the analysis, we contracted all five members of NF-*κ*B family into one node. As the resulting protein interaction network, shown in Figure 3(a), is chordal without weak siblings, functional groups correspond to maximal cliques in the network.

For this network, there are two alternative Tree of Complexes representations: functional group *E *can be connected to either *D *or *C*. The representation that maximizes the number of leaves is shown in Figure 3(b). One can clearly see the interplay between the activators and inhibitors. Proteins p105 and NF-*κ*B participate in the same functional groups and thus follow the same path in the tree. The same is true for the pair of proteins IkB*α *and IkB*β*. The Tree of Complexes captures this by grouping p105 and NF-*κ*B, and IkB*α *and IkB*β*.

### Pheromone signaling pathway

**Figure 4:**
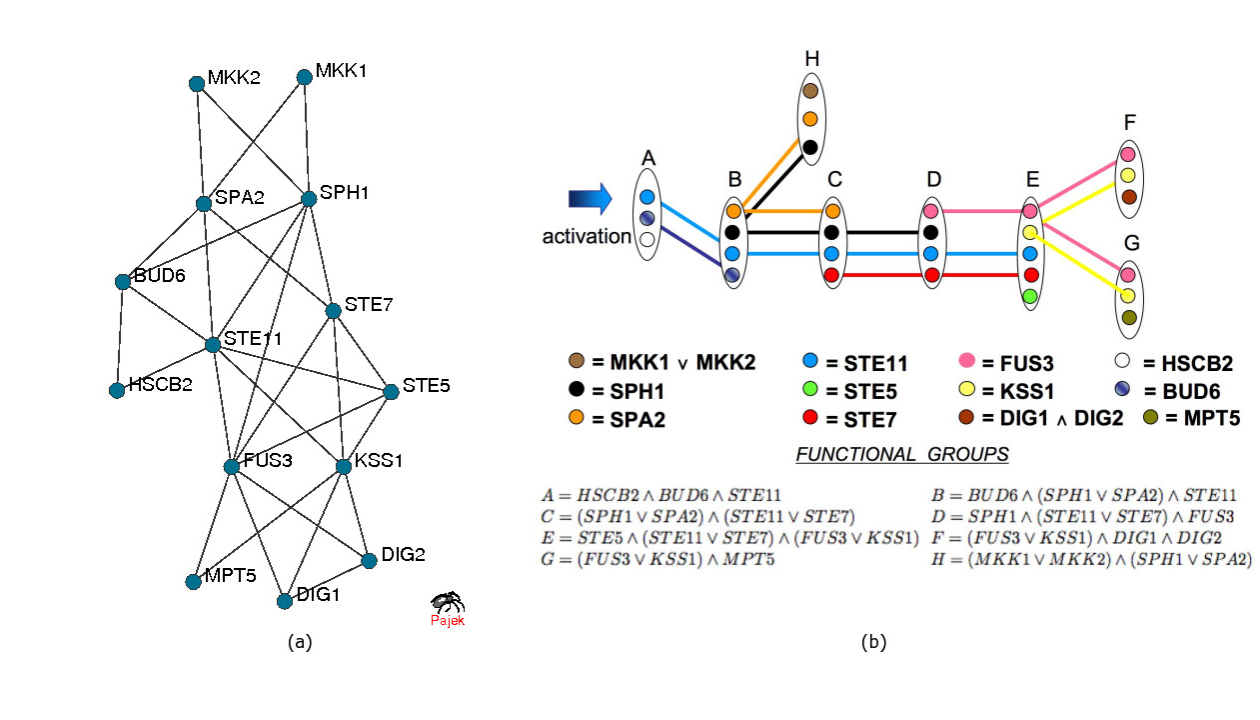
**Pheromone Signaling Pathway**. The pheromone signaling pathway. (a) The network. (b) The Tree of Complexes representation. For the description of the elements of the tree see the text.

The yeast *Saccharomyces cerevisiae *may be present in one of two haploid cell types, which are able to mate. Pheromones released by one type of cell bind to a specific receptor of the other type. This triggers the activation of a scaffold protein-bound mitogen-activated protein kinase (MAPK) cascade and subsequent activation of nuclear proteins that control subsequent cellular events. In a recent paper, Spirin *et al. *[13] identified a subnetwork of proteins involved in this process within a yeast protein interaction network [21]. We analyzed this subnetwork using the COD to see if our method can extract elements of temporal ordering. The subnetwork identified by Spirin *et al. *and its Tree of Complexes representation is given in Figure 4. In this case, the protein network is not chordal. First, the COD method identifies and connects a pair of weak siblings, *MKKl *and *MKK2 *.Then, to transform the network to a chordal graph, three additional edges are added: (*SPH*1, *SPA*2), (*FUS*3, *KSS*1), and (*STE*11, *STE*7). In this case, some functional groups will contain more than one protein complex.

This network admits six different Tree of Complexes representations: (i) functional group *H *can be connected to either *B *or *C*; (ii) any interconnection pattern that spans groups *E*, F, and *G *can be chosen. If we ask for a tree with maximum number of leaves, the number of tree variants is reduced to two (option (i)).

The MAPK cascade module consists of three sequentially acting protein kinases: MAP kinase kinase kinase (STE11) MAP kinase kinase (STE7) and MAP kinase (KSS1, FUS3) [22]. MKKl and MKK2 are two redundant protein kinase kinases (most similar to STE7) [23]. Their redundancy is properly captured by the ∨ (*OR*) in their functional group (H). The MAP kinases KSS1 and FUS3 are two separate kinases both activated by STE7 each of which is essential for a different program: FUS3 – for mating; KSS1 – for the filamentous growth [24]. Once again this is correctly captured by ∨ (*OR*) in groups F and G. STE5 is a scaffold protein of the MAPK module. It recruits MAPK module kinases (STE11, STE7, FUS3). This is consistent with the central position of a functional group containing STE5 in the tree and relative to the paths of STE7, STE11 and FUS3. Finally, nuclear proteins DIG1 and DIG2 (necessary for transcription inhibition, which are regulated by both FUS3 and KSS1) enter at the endpoint (node *F*) in the tree.

## Conclusion

Recent advances in experimental techniques resulted in the accumulation of a vast amount of protein interaction information, which is routinely represented by protein interaction networks. Therefore it is not surprising that increasingly more complex graph-theoretical tools are deployed to analyze protein interaction graphs and extract biologically meaningful patterns.

In general, graphs are not required to have any type of regularity. This makes them a very flexible tool which is able to represent complex relationships. However, this often also makes them computationally hard to deal with, for many problems in graph theory are NP-complete. Frequently graph theoretical problems can be simplified if some restrictions are imposed on the graph. Various restrictions give rise to various graph families. Given a graph family, it is usually very useful to be able to represent it using some kind of a tree. Such tree representation exposes a hierarchical organization that a graph may have, allowing for a structured analysis of it.

In this work we proposed a tree representation for protein interaction graphs called Tree of Complexes representation. Nodes in the Tree of Complexes are functional groups and the tree satisfies the additional condition that functional groups that contain any fixed protein form a connected subgraph. In this way, our representation captures not only the overlap between functional groups but, potentially, also the manner in which proteins enter and leave their enclosing functional groups. We developed a method (together with the corresponding graph-theoretical theory) for efficient identification of such overlapping functional groups and construction of the corresponding Tree of Complexes. In particular, our method differs from other approaches in that it does not attempt to enumerate disjoint complexes but instead identifies and represents relations between overlapping functional groups. Even though the Tree of Complexes representation is not unique, the protein interaction networks that we analyzed admit very few alternative tree topologies. If we ask for a tree topology with a maximum number of leaves, as not to impose an artificial order between functional groups, the number of tree topologies is reduced even further. Thus, in the TNF*α*/NF-*κ*B signalling pathway this results in a unique Tree of Complexes representation and in the pheromone signalling pathway in two very closely related possible Tree of Complexes representations. The nature of high-throughput protein interaction data does not directly imply that this data encodes temporal relations. We demonstrated that our method is frequently capable of discovering such temporal relations. Interestingly, temporal associations can also be implicated in the absence of actual interaction in the data. For example, in the case of the pheromone signaling pathway, our method correctly included KSS1 and FUS3 in the same functional group (treated here as temporal associations), despite the fact that there is no link between KSS1 and FUS3 in the input protein interaction network.

Obviously, there are limitations to deciphering such temporal relations. For example, we cannot provide temporal ordering between different tree branches. Furthermore, if a functional group is not a clique but is represented as a Boolean expression indicating various possibilities for such group, then one can not be sure if these variants are mutually exclusive or if they represent partial information capturing incomplete data. Even in the case when the functional unit forms a clique it is still possible that it contains interactions that are not simultaneous. For example, interactions between pairs (A, B), (B, C) and (C, A) are represent as a three-vertex clique with nodes A, B, C and thus cannot be distinguished from a trimer (A, B, C). Such coincidences are less likely for larger cliques.

Although our algorithm is not guaranteed to produce the Tree of Complexes representation for every possible protein interaction network, the algorithm will succeed for a broad family of graphs, which includes chordal graphs (and thus interval graphs) and cographs. Currently, our method can be applied to protein interaction networks that do not contain long (longer than four node) chordless cycles. As a consequence, it is more appropriate for analyzing dedicated subnetworks or modules than large protein interaction networks, which are expected to contain such long cycles. We distinguish between two different types of problematic networks for our method. First type includes networks for which imposing a temporal order that encompasses all functional groups in the network is meaningless. Second type includes networks for which such order is meaningful, but the assumption that the overlap between functional groups has a tree-like structure is not valid. We plan to extend our approach to deal with networks of the second type by utilizing graph-theoretical tools developed for other specialized graph families, such as arc-intersection graphs.

Another issue that requires further investigation is the presence of noise in high-throughput protein interaction networks and its effect on the Tree of Complexes representation. While our method deals to some extent with false negatives, through its edge addition procedure, the issue of false positives is not addressed. We plan to explore alternative graph modification procedures that will incorporate both false negative and false positive interactions.

## Methods

### Computing clique trees

We use an elegant and efficient strategy for chordal graph recognition outlined in [19]. We use an algorithm from [25] to compute all clique trees for a chordal graph.

### A compact boolean representation of functional groups

Recall that a functional group corresponds to a maximal clique in the modified protein interaction network, with modifications being addition of edges between every pair of weak siblings and then addition of edges that eliminate all the squares in the graph. The edge addition is such that no maximal clique in the modified graph contains an induced *P*_4 _formed entirely by the added edges. Following lemma guarantees that every functional group is a cograph and therefore admits a compact Boolean representation.

**Lemma **For every functional group, a subgraph of the original graph induced by the members of the group contains an induced *P*_4 _if and only if the set of edges added by our algorithm contains an induced *P*_4_.

**Figure 5:**
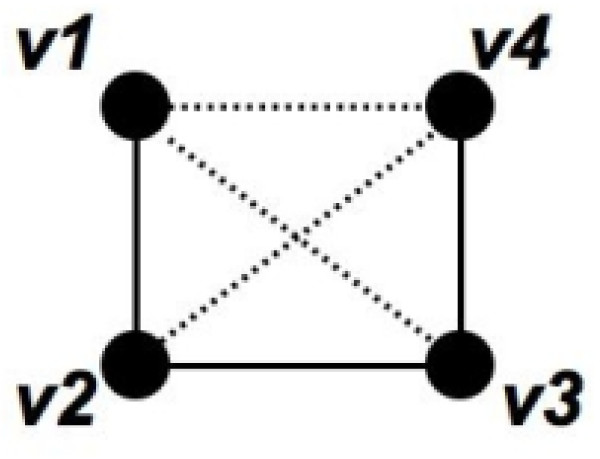
**P4**. A *P*_4 _in the subgraph induced by the members of a functional group corresponds to a *P*_4 _in the set of added edges. Solid lines correspond to the original edges and dashed lines correspond to the added edges.

**Proof **The argument follows from Figure 5. Indeed, (*v*_1_, *v*_2_, *v*_3_, *v*_4_) is a *P*_4 _in the original graph if and only if (*v*_3_, *v*_1_, *v*_4_, *v*_2_) is a *P*_4 _formed by the added edges.

### Edge addition

We use a reduction to the Minimum Vertex Cover problem to find all minimal sets of up to *k *edges that eliminate all the squares in the graph.

Each eliminating set of edges, *S *= {*e*_1_,..., *e*_*r*_}, is assigned a cost:



where sim(*e*_*i*_) takes values between 1.0 and 0.0, and measures our confidence in adding *e*_*i *_to the graph. Since the addition of *e*_*i *_= (*u*_*i*_, *v*_*i*_) implies an interaction or functional equivalence between proteins *u*_*i *_and *v*_*i*_, we chose sim(*e*_*i*_) to be the amount of overlap between the neighborhoods of *u*_*i *_and *v*_*i*_, i.e., sim(*e*_*i*_) = , where  (*v*_*i*_) denotes a set of neighbors of node *v*_*i *_in the graph. Intuitively, sim(*e*_*i*_) measures how close *u*_*i *_and *v*_*i *_are to being a pair of weak siblings. If *u*_*i *_and *v*_*i *_have the same neighborhoods then sim(*e*_*i*_) = 1.0; as the overlap between the neighborhoods decreases, sim(*e*_*i*_) goes to 0.0. Then, we pick an edge set with the minimum cost from all the edge sets that do not form a *P*_4_, which is entirely contained in one of the maximal cliques of the modified graph. The last requirement is necessary to ensure that each functional group is a cograph.

### Reduction to the minimum vertex cover

**Figure 6:**
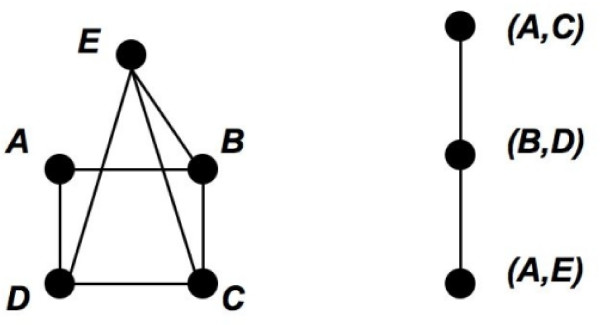
**VC Reduction**. A graph and a corresponding "square coverage graph".

A square in a graph can be eliminated by adding one or both of its diagonals (chords) to the graph. For example, a graph in Figure 6 has two squares: (*A, B, C, D*) and (*A, B, E, D*). Note that (*B, C, D, E*) is not a square as one of its diagonals, (*C, E*), is an edge in the graph. The square (*A, B, C, D*) can be eliminated if either edge (*A, C*) or (*B, D*) is added to the graph. Furthermore, a single diagonal can eliminate more than one square. For example, the diagonal (*B, D*)eliminates both squares. We are interested in finding all minimal sets of diagonals of size up to *k *that eliminate all the squares in the graph.

We reduce the above problem to the Minimum Vertex Cover problem. The squares in the original graph become edges and diagonals become vertices in the new graph. Thus the original graph is transformed to a "square coverage" graph, which in turn serves as an input to the Minimum Vertex Cover problem. In the Minimum Vertex Cover problem we are given a graph and are asked to find the smallest set of vertices that cover all the edges in the graph. An edge is covered if at least one of its end points is selected. Coming back to our example, it can be easily seen that {(B, D)} is the minimum vertex cover (Figure 6). Although the Minimum Vertex Cover problem is an NP-hard problem, if the size of the optimum solution is small an efficient algorithm can be obtained. In other words the Minimum Vertex Cover problem is fixed parameter tractable. We use an *O*(2^*k*^(*n *+ *m*)) algorithm [26] to identify all minimal sets of edges of size up to *k *that eliminate all the squares in the graph.

### Graphs that have tree of complexes representation

The COD method is not guaranteed to produce the Tree of Complexes representation for every possible protein interaction network. How can a family of graphs that admit Tree of Complexes representation be characterized? First, we argue that chordal graphs belong to this family. It can be shown that addition of edges that connect weak siblings does not introduce chordless cycles to the graph. Therefore, after all weak siblings are connected the graph is still chordal and thus admits Tree of Complexes representation. Next, we argue that cographs admit Tree of Complexes representation. Our methods eliminates all the squares in the graph, unless every possible set of eliminating edges forms a *P*_4_, which is entirely contained in one of the maximal cliques of the modified graph. It can be shown that the latter case is possible only when the original graph contains a *P*_4_, and thus is not a cograph. We conjecture that graphs that admit Tree of Complexes representation are exactly those graphs that admit a clique tree representation, with the nodes being maximal cographs rather than maximal cliques.

## Competing interests

The author(s) declare that they have no competing interests.

## Authors' contributions

EZ implemented the algorithm implementation, and participated in design of experiments, analysis of results, and drafting of the manuscript. KG and RJ participated in design of experiments, analysis of results, and revising of the manuscript. TP conceived the project and participated in the design of experiments, analysis of results, and drafting of the manuscript.
